# Self-Harm Events and Suicide Deaths Among Autistic Individuals in Ontario, Canada

**DOI:** 10.1001/jamanetworkopen.2023.27415

**Published:** 2023-08-08

**Authors:** Meng-Chuan Lai, Natasha R. Saunders, Anjie Huang, Azmina Artani, Andrew S. Wilton, Juveria Zaheer, Stephanie H. Ameis, Hilary K. Brown, Yona Lunsky

**Affiliations:** 1 Campbell Family Mental Health Research Institute, Centre for Addiction and Mental Health, Toronto, Ontario, Canada; 2 Department of Psychiatry, The Hospital for Sick Children, Toronto, Ontario, Canada; 3 Department of Psychiatry, Temerty Faculty of Medicine, University of Toronto, Toronto, Ontario, Canada; 4 Autism Research Centre, Department of Psychiatry, University of Cambridge, Cambridge, United Kingdom; 5 Department of Psychiatry, National Taiwan University Hospital and College of Medicine, Taipei, Taiwan; 6 Child Health Evaluative Sciences, SickKids Research Institute, Toronto, Ontario, Canada; 7 Department of Paediatrics, Temerty Faculty of Medicine, University of Toronto, Toronto, Ontario, Canada; 8 ICES, Ontario, Canada; 9 Leong Centre for Healthy Children, University of Toronto, Toronto, Ontario, Canada; 10 Department of Health and Society, University of Toronto Scarborough, Toronto, Ontario, Canada

## Abstract

**Importance:**

Reasons for elevated suicide risks among autistic people are unclear, with insufficient population-based research on sex-specific patterns to inform tailored prevention and intervention.

**Objectives:**

To examine sex-stratified rates of self-harm events and suicide death among autistic individuals compared with nonautistic individuals, as well as the associated sociodemographic and clinical risk factors.

**Design, Setting, and Participants:**

This population-based matched-cohort study using linked health administrative databases in Ontario, Canada included all individuals with physician-recorded autism diagnoses from April 1, 1988, to March 31, 2018, each matched on age and sex to 4 nonautistic individuals from the general population. Self-harm events resulting in emergency health care from April 1, 2005, to December 31, 2020, were examined for one cohort, and death by suicide and other causes from April 1, 1993, to December 31, 2018, were examined for another cohort. Statistical analyses were conducted between October 2021 and June 2023.

**Exposure:**

Physician-recorded autism diagnoses from 1988 to 2018 from health administrative databases.

**Main Outcomes and Measures:**

Autistic and nonautistic individuals who were sex stratified a priori were compared using Andersen-Gill recurrent event models on self-harm events, and cause-specific competing risk models on death by suicide or other causes. Neighborhood-level income and rurality indices, and individual-level broad diagnostic categories of intellectual disabilities, mood and anxiety disorders, schizophrenia spectrum disorders, substance use disorders, and personality disorders were covariates.

**Results:**

For self-harm events (cohort, 379 630 individuals; median age at maximum follow-up, 20 years [IQR, 15-28 years]; median age of first autism diagnosis claim for autistic individuals, 9 years [IQR, 4-15 years]; 19 800 autistic females, 56 126 autistic males 79 200 nonautistic females, and 224 504 nonautistic males), among both sexes, autism diagnoses had independent associations with self-harm events (females: relative rate, 1.83; 95% CI, 1.61-2.08; males: relative rate, 1.47; 95% CI, 1.28-1.69) after accounting for income, rurality, intellectual disabilities, and psychiatric diagnoses. For suicide death (cohort, 334 690 individuals; median age at maximum follow-up, 19 years [IQR, 14-27 years]; median age of first autism diagnosis claim for autistic individuals, 10 years [IQR, 5-16 years]; 17 982 autistic females, 48 956 autistic males, 71 928 nonautistic females, 195 824 nonautistic males), there was a significantly higher crude hazard ratio among autistic females (1.98; 95% CI, 1.11-3.56) and a nonsignificantly higher crude hazard ratio among autistic males (1.34; 95% CI, 0.99-1.82); the increased risks were associated with psychiatric diagnoses.

**Conclusions and Relevance:**

This cohort study suggests that autistic individuals experienced increased risks of self-harm events and suicide death. Psychiatric diagnoses were significantly associated with the increased risks among both sexes, especially for suicide death, and in partially sex-unique ways. Autism-tailored and autism-informed clinical and social support to reduce suicide risks should consider multifactorial mechanisms, with a particular focus on the prevention and timely treatment of psychiatric illnesses.

## Key Points

**Question:**

What are the sex-stratified rates of self-harm events and suicide death among autistic individuals vs nonautistic individuals and the associated sociodemographic and clinical risk factors?

**Findings:**

In this cohort study including 379 630 individuals regarding self-harm findings and 334 690 individuals regarding suicide death findings in Ontario, Canada, autistic females had an 83% increased risk and autistic males had a 47% increased risk of self-harm compared with nonautistic individuals, when accounting for neighborhood income and rurality, intellectual disabilities, and psychiatric diagnoses. The crude hazard ratio showed that autistic females had a 98% increased risk and autistic males had a 34% increased risk of suicide death, but these increases were associated with psychiatric diagnoses.

**Meaning:**

This study suggests that psychiatric diagnoses were significantly associated with risks of self-harm and especially suicide among autistic females and males.

## Teaser

This cohort study including all individuals with autism diagnoses recorded in health administrative databases in Ontario examines sex-stratified rates of self-harm events and suicide death among autistic individuals vs nonautistic individuals, as well as associated sociodemographic and clinical risk factors.

## Introduction

Autistic individuals experience higher mortality than nonautistic individuals across natural and unnatural causes.^1^ Among potentially preventable causes, suicide death and precursory self-harm events^2^ occur at higher rates among autistic individuals than nonautistic individuals.^3,4,5,6,7,8^ Meta-analyses show that autistic individuals have over 3-fold greater odds than nonautistic individuals of experiencing self-injurious behavior, suicidal ideation, suicide attempt, or suicide death.^4^ One-fourth of autistic young people experience suicidal ideation and 8.3% experience suicide attempts.^3^

Reasons for increased suicide risks among autistic people are unclear. Reported risk factors include adverse childhood experiences, poor social connection, and psychiatric illnesses (eg, depression and psychosis).^3,6^ High heterogeneity and inconsistent demographic correlates across studies^3,4^ suggest that rates of suicide-related events and, potentially, rates and kinds of suicide risk and protective factors, vary across demographics and jurisdictions. Population-based studies offer critical insights about suicide risks among autistic people and region-specific variations by sociocultural and health systems factors. So far, there are only a small number of such studies, from the US,^9,10,11,12,13^ Sweden,^14,15,16,17^ Denmark,^18^ Finland,^19^ Taiwan,^20,21^ and the UK.^22^

Suicide risk patterns can be sex-specific, with males more likely to die by suicide than females, and females experiencing more self-harm, suicidal ideation, and suicide attempts than males.^23,24^ Among autistic individuals, recent Scandinavian and US population-based studies found that female sex assigned at birth (hereafter referred to as sex) is associated with greater self-harm events and suicidality overall,^10,11,14,15,18,19^ although studies in other countries or with more confined age groups did not observe this trend.^13,20,21,22^ This pattern implies unique modulation regarding suicide risks by sex-related factors (eg, differences in vulnerability to psychiatric illnesses)^25^ and/or gender-related factors (eg, gender diversity status)^26^ among autistic people.^3,6^ Simply adjusting for sex (or gender), but not considering stratification, in investigating suicide risks among autistic people^17^ might miss mechanistic insights. Sex stratification is essential to unveil unique suicide risks among autistic individuals, especially because females have been underrepresented in autism research for decades.^27^

In this, to our knowledge, first Canadian population-based study including all individuals with autism diagnoses recorded in health administrative databases in Ontario, we add to the growing population-based research to investigate sex-stratified rates of self-harm events (leading to emergency health care) and suicide death among autistic or nonautistic people as well as associated sociodemographic and clinical factors.

## Methods

### Study Design and Data Sources

We conducted a population-based, matched-cohort study in Ontario, the largest Canadian province (14.7 million residents), following the Strengthening the Reporting of Observational Studies in Epidemiology (STROBE) reporting guideline. In Ontario’s single-payer universal health care system, residents receive publicly funded hospital inpatient, emergency department, and outpatient physician services. Data sets were linked using unique encoded identifiers and analyzed at ICES, an independent nonprofit research institute whose legal status under Ontario’s health information privacy law allows it to collect and analyze health care and demographic data, without consent, for health system evaluation and improvement. The use of these data is authorized under section 45 of Ontario’s Personal Health Information Protection Act and does not require review by a research ethics board.

We identified all autistic individuals, of all ages, with physician-recorded autism diagnoses from April 1, 1988, to March 31, 2018, using the following databases: Canadian Institute for Health Information Discharge Abstract Database, Same Day Surgery Database, National Ambulatory Care Reporting System (NACRS), Ontario Mental Health Reporting System, and Ontario Health Insurance Plan Database (OHIP) (eTable 1 in Supplement 1). Autistic individuals were identified when autism diagnoses were recorded at 2 or more outpatient physician visits, 1 or more emergency department visit or same-day surgery, or 1 or more hospitalization, using codes from the *International Classification of Diseases, Ninth Revision* (code 299), *International Statistical Classification of Diseases and Related Health Problems, Tenth Revision* (*ICD-10*) (codes F84.0, F84.1, F84.3, F84.5, F84.8, and F84.9), or *Diagnostic and Statistical Manual of Mental Disorders* (Fourth Edition) (code 299), as a fixed-effect variable. This previously used algorithm^28^ has excellent specificity and moderate sensitivity.^29^

For self-harm events and suicide death we had different accrual and follow-up periods owing to data availability (see Outcome Measures). Therefore, we created 2 largely overlapping cohorts of autistic individuals (84.7% [65 513 of 77 351] in both cohorts) to separately evaluate the 2 outcomes. For both cohorts, the accrual period began at a person’s 10th birthday, considering the rare incidence of these outcomes at younger than 10 years of age.^17,18^ An autistic individual was matched to 4 nonautistic individuals (who never had an autism diagnosis recorded) on age and sex to create the comparison group. Individuals were excluded if they had missing age or sex data (assumed completely at random; none was excluded), were older than 105 years, were not an Ontario resident or died at or before cohort entry, lost OHIP coverage for more than 90 days in the 2 years before cohort entry, or had no health care contact in the 5 years before cohort entry.

### Outcome Measures

Self-harm events leading to emergency health care was ascertained from the NACRS with the *ICD-10* codes X60 to X84 (intentional self-harm) and codes Y10 to Y34 (events of undetermined intent), treated as recurrent events. Considering NACRS data availability (from 2002) and a lookback period to capture previous health system use, the accrual period was April 1, 2005, to March 31, 2020, with maximum follow-up until December 31, 2020. Death by suicide or by other causes was ascertained from the Ontario Registrar General Vital Statistics—Deaths (ORGD) registry and Registered Persons Database (RPDB). Considering ORGD data availability, the accrual period was April 1, 1993, to March 31, 2018, with maximum follow-up until December 31, 2018. For both cohorts, the observation period was terminated at death (from any cause), end of follow-up, or with lost OHIP coverage for more than 6 months.

### Sociodemographic and Clinical Variables

Age, sex, and postal code were obtained from the RPDB and linked to Statistics Canada’s Postal Code Conversion File version 2016^30^ to ascertain neighborhood-level median income (quintiles) and rurality (rural residence if community size ≤10 000) at cohort entry for each individual.^31^ Lifetime intellectual disability diagnoses were ascertained as a fixed-effect variable when recorded at 2 or more outpatient physician visits, 1 or more emergency department visit or same-day surgery, or 1 or more hospitalization until the end of follow-up.^32^ Lifetime psychiatric diagnoses categories were ascertained until the end of follow-up using algorithms from previous Ontario-based studies^33,34,35,36^ (eTable 2 in Supplement 1), all modeled as time-varying variables: (1) mood and anxiety disorders, (2) schizophrenia spectrum and psychotic disorders, (3) substance-related and addictive disorders, and (4) personality disorders.

### Statistical Analysis

All analyses were sex stratified. Within each sex, sociodemographic characteristics, lifetime prevalence of clinical diagnoses, incidence of death (by suicide or other causes), and self-harm events were described separately for autistic groups and age-matched nonautistic groups (Table 1 and Table 2).^37^

**Table 1.** Description of the Sex-Stratified, Age-Matched Cohort for Self-Harm Events^a^

Characteristic	Individuals, No. (%)		Standardized difference^b^	Individuals, No. (%)		Standardized difference^b^
	Autistic females (n = 19 800)	Nonautistic females (n = 79 200)		Autistic males (n = 56 126)	Nonautistic males (n = 224 504)	
Age of first autism diagnosis claim, median (IQR), y	12 (6-25)	NA	NA	8 (4-14)	NA	NA
Age at maximum follow-up, median (IQR), y	22 (16-38)	22 (16-38)	0	19 (14-27)	19 (14-26)	0.01
Follow-up duration, median (IQR), y	11 (6-16)	11 (5-16)	0	9 (5-16)	9 (5-16)	0.01
Neighborhood income quintile						
Q1 (lowest)	4187 (21.1)	14 957 (18.9)	0.06	11 478 (20.5)	41 107 (18.3)	0.05
Q2	3924 (19.8)	14 791 (18.7)	0.03	10 933 (19.5)	41 081 (18.3)	0.03
Q3	3805 (19.2)	15 912 (20.1)	0.02	11 019 (19.6)	45 398 (20.2)	0.01
Q4	3999 (20.2)	16 436 (20.8)	0.01	11 436 (20.4)	47 749 (21.3)	0.02
Q5 (highest)	3802 (19.2)	16 823 (21.2)	0.05	11 036 (19.7)	48 408 (21.6)	0.05
Missing	83 (0.4)	281 (0.4)	0.01	224 (0.4)	761 (0.3)	0.01
Rural index						
Urban	17 775 (89.8)	69 948 (88.3)	0.05	50 417 (89.8)	198 666 (88.5)	0.04
Rural	2002 (10.1)	9185 (11.6)	0.05	5635 (10.0)	25 670 (11.4)	0.05
Missing	23 (0.1)	67 (0.1)	0.01	74 (0.1)	168 (0.1)	0.02
Diagnoses ever recorded						
Intellectual disabilities	3216 (16.2)	239 (0.3)	0.6	7943 (14.2)	1057 (0.5)	0.54
Mood and anxiety disorders	14 820 (74.8)	41 216 (52.0)	0.49	36 265 (64.6)	82 674 (36.8)	0.58
Schizophrenia spectrum and other psychotic disorders	3097 (15.6)	1376 (1.7)	0.51	7833 (14.0)	3644 (1.6)	0.47
Substance-related and addictive disorders	1836 (9.3)	4183 (5.3)	0.15	4346 (7.7)	14 041 (6.3)	0.06
Personality disorders	2695 (13.6)	2180 (2.8)	0.4	5373 (9.6)	4361 (1.9)	0.33
Ever had self-harm events leading to emergency health care	1586 (8.0)	1762 (2.2)	0.26	2080 (3.7)	2627 (1.2)	0.16
Age at first self-harm event leading to emergency health care, if present, median (IQR), y	17.9 (15.4-25.6)	17.5 (15.4-22.2)	0.08	20.1 (16.6-26.5)	19.6 (16.7-24.8)	0.04

Abbreviations: NA, not applicable; Q, quintile.

^a^ Accrual period from 10th birthday (or from April 1, 2005, for those older than 10 years at this date) to March 31, 2020. Most autistic individuals, across sexes, were identified via autism diagnoses recorded in outpatient and/or inpatient settings; 2.4% of autistic females (481 of 19 800) and 2.4% of autistic males (1370 of 56 126) were identified exclusively via records in emergency department visits.

^b^ Standardized difference of 0.1 or more implies clinical meaningfulness.^37^

**Table 2.** Description of the Sex-Stratified, Age-Matched Cohort for Death by Suicide or Other Causes^a^

Characteristic	Individuals, No. (%)		Standardized difference^b^	Individuals, No. (%)		Standardized difference^b^
	Autistic females (n = 17 982)	Nonautistic females (n = 71 928)		Autistic males (n = 48 956)	Nonautistic males (n = 195 824)	
Age of first autism diagnosis claim, median (IQR), y	13 (7-30)	NA	NA	9 (4-15)	NA	NA
Age at maximum follow-up, median (IQR), y	22 (15-41)	21 (15-40)	0.01	19 (14-26)	19 (14-25)	0.01
Follow-up duration, median (IQR), y	11 (5-21)	10 (5-20)	0.04	9 (5-16)	9 (4-15)	0.03
Neighborhood income quintile						
Q1 (lowest)	3671 (20.4)	13 713 (19.1)	0.03	9849 (20.1)	36 145 (18.5)	0.04
Q2	3527 (19.6)	13 354 (18.6)	0.03	9502 (19.4)	35 825 (18.3)	0.03
Q3	3416 (19.0)	14 181 (19.7)	0.02	9601 (19.6)	39 371 (20.1)	0.01
Q4	3528 (19.6)	14 786 (20.6)	0.02	9876 (20.2)	41 601 (21.2)	0.03
Q5 (highest)	3501 (19.5)	14 903 (20.7)	0.03	9573 (19.6)	41 422 (21.2)	0.04
Missing	339 (1.9)	991 (1.4)	0.04	555 (1.1)	1460 (0.7)	0.04
Rural index						
Urban	15 727 (87.5)	62 028 (86.2)	0.04	43 334 (88.5)	170 708 (87.2)	0.04
Rural	2140 (11.9)	9628 (13.4)	0.04	5285 (10.8)	24 747 (12.6)	0.06
Missing	115 (0.6)	272 (0.4)	0.04	337 (0.7)	369 (0.2)	0.08
Diagnoses ever recorded						
Intellectual disabilities	3048 (17.0)	238 (0.3)	0.62	7595 (15.5)	1036 (0.5)	0.57
Mood and anxiety disorders	13 306 (74.0)	35 576 (49.5)	0.52	31 553 (64.5)	68 774 (35.1)	0.61
Schizophrenia spectrum and other psychotic disorders	2736 (15.2)	1216 (1.7)	0.5	6861 (14.0)	2893 (1.5)	0.48
Substance-related and addictive disorders	1540 (8.6)	3597 (5.0)	0.14	3630 (7.4)	11 500 (5.9)	0.06
Personality disorders	2276 (12.7)	1812 (2.5)	0.39	4676 (9.6)	3635 (1.9)	0.34
Death by suicide in this 25 study-years, individuals, No. (incidence per 100 000 population)	17 (95/100 000)	33 (46/100 000)	0.02	57 (116/100 000)	163 (83/100 000)	0.01
Age at death by suicide, if present, median (IQR), y	28.4 (21.0-41.3)	26.9 (18.2-50.8)	0.01	25.4 (21.0-30.8)	26.4 (19.8-46.6)	0.10
Death by nonsuicide causes in this 25 study-years, individuals, No. (incidence per 100 000 population)	1762 (9799/100 000)	5836 (8114/100 000)	0.06	1671 (3413/100 000)	5073 (2591/100 000)	0.05

Abbreviations: NA, not applicable; Q, quintile.

^a^ Accrual period from 10th birthday (or from April 1, 1993, for those older than 10 years at this date) to March 31, 2018. Most autistic individuals, across sexes, were identified via autism diagnoses recorded in outpatient and/or inpatient settings; 2.5% of autistic females (449 of 17 982) and 2.5% of autistic males (1240 of 48 956) were identified exclusively via records in emergency department visits.

^b^ Standardized difference of 0.1 or more implies clinical meaningfulness.^37^

For self-harm events, we demonstrated the cumulative risks by sex and autistic or nonautistic groups using cumulative mean function curves (Figure). We used the Andersen-Gill recurrent event model, a generalization of the Cox proportional hazards regression model that models the intensity or rate of self-harm over time,^38^ to examine the association between autism diagnosis and self-harm event(s), using the robust sandwich variance estimation to account for multiple self-harm events in the same individual.^39^ We first ran an unadjusted model to ascertain a crude relative rate (RR) of the association of autism diagnosis and self-harm, then adjusted for income and rurality, intellectual disabilities, and each psychiatric diagnosis one at a time to observe the change in RR. For all models we further adjusted for previous self-harm event(s) as a time-varying cumulative count covariate.^40^ Eventually, a final multivariable model with forward selection determined the most important correlates for self-harm events across the cohort.

**Figure.**
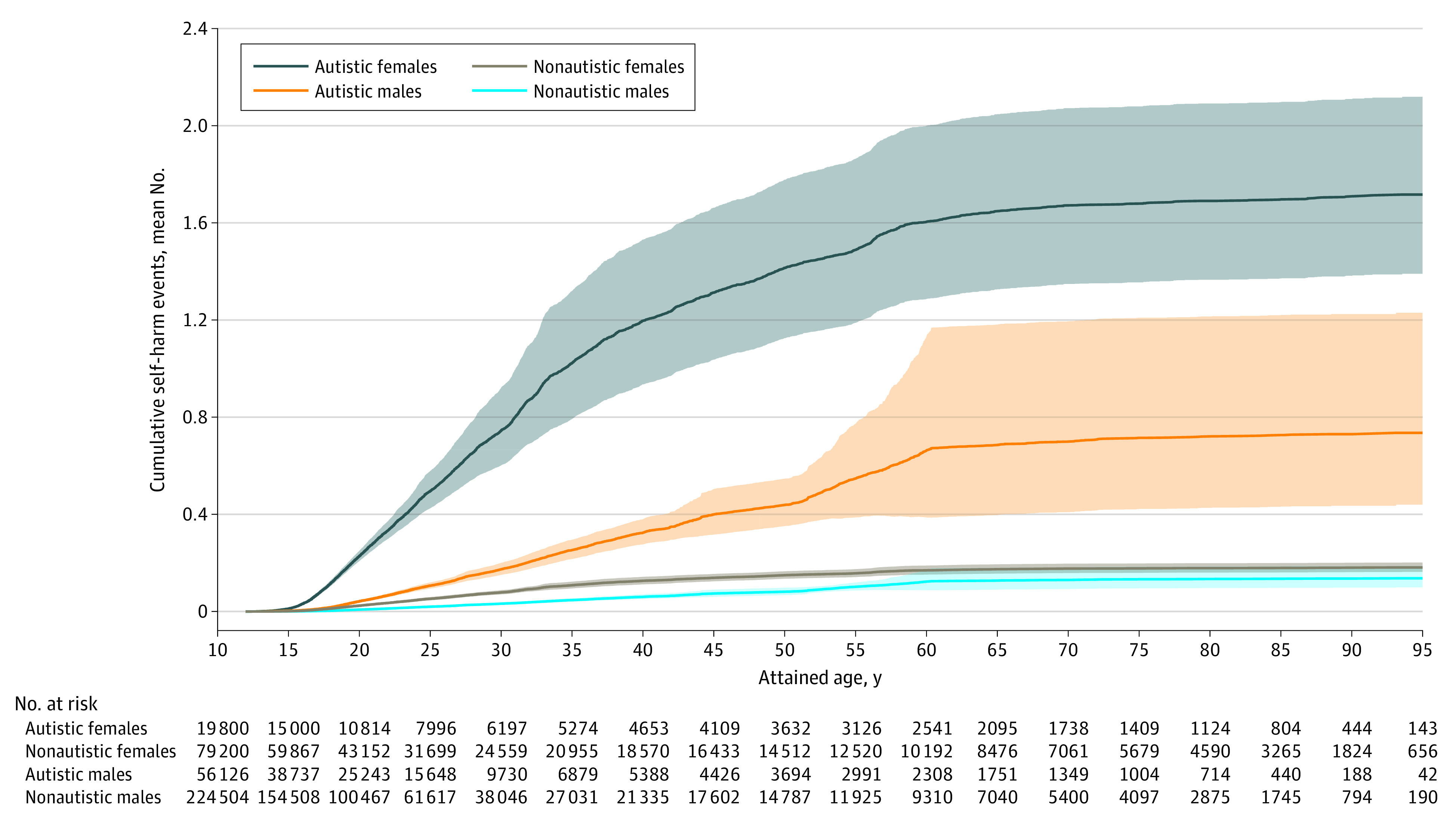
Cumulative Mean Function Curves for Self-Harm Events by Group Estimated mean cumulative numbers of self-harm events by attained age are illustrated separately for autistic females, autistic males, nonautistic females, and nonautistic males. Shaded areas indicate 95% CIs of the estimated means. Numbers of individuals at risk in each group at intervals along the x-axis were calculated to indicate those who were not censored at the attained age.

For suicide death, we used cause-specific competing risk models on death by suicide or other causes.^41^ We first ran an unadjusted model to estimate a crude hazard ratio (HR) of the association between autism diagnosis and death by suicide and by other causes, then adjusted for income and rurality, intellectual disabilities, and each psychiatric diagnosis one at a time to observe the change in HR. A final multivariable model with forward selection determined the most important correlates of suicide death across the cohort. Analyses were performed with SAS, version 9.4 (SAS Institute Inc) (eAppendix in Supplement 1). Statistical significance was determined by the 95% CI of RR or HR not including 0.

## Results

### Self-Harm Events

The self-harm events cohort included 379 630 individuals (median age at maximum follow-up, 20 years [IQR, 15-28 years]; median age of first autism diagnosis claim for autistic individuals, 9 years [IQR, 4-15 years]; 19 800 autistic females, 79 200 nonautistic females; 56 126 autistic males, 224 504 nonautistic males) followed up for self-harm events from 2005 to 2020 (Table 1). Ascertaining all autistic individuals in Ontario resulted in an older age distribution of the female group than the male group. Autistic females showed the highest cumulative risk of self-harm events (estimated mean cumulative number of self-harm events, 1.7 by an attained age of 90 years), followed by autistic males (0.7), nonautistic females (0.2), and nonautistic males (0.1) (Figure). For those experiencing self-harm, the median age at the first event was 17.9 years (IQR, 15.4-25.6 years) for autistic females, 20.1 years (IQR, 16.6-26.5 years) for autistic males, 17.5 years (IQR, 15.4-22.2 years) for nonautistic females, and 19.6 years (IQR, 16.7-24.8 years) for nonautistic males.

Among females (Table 3), self-harm risk was significantly higher for autistic individuals than nonautistic individuals (crude RR, 9.46; 95% CI, 7.83-11.43). The pattern did not change when adjusted for neighborhood income and rurality but was attenuated when adjusted separately for intellectual disabilities and each psychiatric diagnosis—although the risks remained significantly higher among autistic females than nonautistic females. Further adjusting for cumulative self-harm event(s) attenuated all RRs but they all remained significant (range, 4.00-6.76).

**Table 3.** Risks of Self-Harm Events and Risks of Suicide Death and Other Causes of Death Among Autistic vs Nonautistic Groups, Stratified by Sex Assigned at Birth

Risk model	Recurrent event Andersen-Gill models on self-harm events, RR (95% CI)				Cause-specific competing risk models on death by suicide and by other causes, HR (95% CI)			
	Autistic vs nonautistic females	Autistic vs nonautistic females, further adjusted for previous self-harm events as cumulative counts	Autistic vs nonautistic males	Autistic vs nonautistic males, further adjusted for previous self-harm events as cumulative counts	Autistic vs nonautistic females: suicide death	Autistic vs nonautistic females: other causes of death	Autistic vs nonautistic males: suicide death	Autistic vs nonautistic males: other causes of death
Crude	9.46 (7.83-11.43)	8.01 (6.97-9.22)	5.38 (4.29-6.76)	4.71 (4.23-5.25)	1.98 (1.11-3.56)	1.08 (1.03-1.14)	1.34 (0.99-1.82)	1.15 (1.09-1.22)
Adjusted for income and rurality only	9.47 (7.82-11.48)	7.98 (6.94-9.18)	5.31 (4.28-6.60)	4.69 (4.21-5.22)	1.99 (1.11-3.58)	1.08 (1.02-1.14)	1.36 (1.01-1.85)	1.14 (1.07-1.20)
Adjusted for intellectual disabilities only	5.41 (4.71-6.22)	5.28 (4.60-6.06)	3.25 (2.82-3.75)	3.18 (2.77-3.64)	2.35 (1.28-4.32)	1.02 (0.96-1.07)	1.62 (1.18-2.23)	1.00 (0.94-1.06)
Adjusted for mood and anxiety disorders only	6.25 (5.11-7.65)	5.16 (4.47-5.97)	3.40 (2.65-4.37)	2.94 (2.63-3.29)	1.52 (0.84-2.75)	1.06 (1.00-1.12)	0.99 (0.73-1.35)	1.10 (1.04-1.16)
Adjusted for schizophrenia spectrum and other psychotic disorders only	4.83 (4.18-5.58)	4.59 (3.99-5.28)	2.78 (2.36-3.27)	2.64 (2.28-3.06)	1.17 (0.60-2.30)	1.03 (0.98-1.09)	0.69 (0.48-0.99)	1.02 (0.97-1.08)
Adjusted for substance-related and addictive disorders only	7.74 (6.55-9.13)	6.76 (5.92-7.73)	4.87 (3.89-6.09)	4.26 (3.83-4.75)	1.84 (1.02-3.30)	1.07 (1.01-1.13)	1.27 (0.94-1.72)	1.13 (1.07-1.19)
Adjusted for personality disorders only	4.39 (3.82-5.05)	4.00 (3.53-4.53)	3.25 (2.81-3.75)	3.04 (2.73-3.40)	1.03 (0.55-1.96)	1.06 (1.00-1.12)	1.05 (0.76-1.44)	1.11 (1.05-1.18)

Abbreviations: HR, hazard ratio; RR, relative rate.

Self-harm risk was also significantly higher for autistic males than nonautistic males (crude RR, 5.38; 95% CI, 4.29-6.76) (Table 3). The pattern did not change when adjusted for income and rurality but was attenuated when adjusted separately for intellectual disabilities and each psychiatric diagnosis—all remained significantly higher among autistic males than nonautistic males. Further adjusting for cumulative self-harm event(s) attenuated the RRs and they all remained significant (range, 2.64-4.26).

The final models (Table 4) showed that, for both females and males, autism, neighborhood income, rurality, intellectual disabilities, all 4 psychiatric diagnoses, and cumulative self-harm event(s) were significant correlates. Autism had independent associations with self-harm events (females: RR, 1.83; 95% CI, 1.61-2.08; males: RR, 1.47; 95% CI, 1.28-1.69) even after accounting for sociodemographic factors (varied directions of associations), intellectual disabilities (associated with increased risks), and psychiatric diagnoses (all associated with increased risks).

**Table 4.** Final Models for Correlates of Self-Harm Events and Suicide Death, by Cohort and Sex Assigned at Birth^a^

Correlate	Self-harm events, RR (95% CI)^b^		Suicide death, HR (95% CI)^c^	
	Females	Males	Females	Males
Autism^d^	1.83 (1.61-2.08)	1.47 (1.28-1.69)	0.89 (0.45-1.77)	0.82 (0.58-1.17)
Neighborhood income quintile				
Q2 vs Q1	1.08 (0.87-1.33)	0.88 (0.73-1.06)	NI	NI
Q3 vs Q1	0.96 (0.78-1.17)	0.63 (0.55-0.73)		
Q4 vs Q1	0.84 (0.69-1.03)	0.87 (0.70-1.08)		
Q5 vs Q1	0.76 (0.62-0.95)	0.68 (0.57-0.80)		
Rural index (rural vs urban)	1.33 (1.06-1.66)	1.37 (1.21-1.56)	1.58 (0.79-3.17)	2.23 (1.65-3.02)
Intellectual disabilities	2.05 (1.66-2.55)	2.15 (1.80-2.57)	0.21 (0.05-0.94)	0.28 (0.14-0.57)
Mood and anxiety disorders	6.96 (6.22-7.79)	4.49 (4.10-4.92)	3.78 (1.58-9.06)	2.31 (1.67-3.20)
Schizophrenia spectrum and other psychotic disorders	2.18 (1.82-2.60)	2.17 (1.86-2.51)	2.80 (1.26-6.21)	3.81 (2.57-5.64)
Substance-related and addictive disorders	3.06 (2.58-3.64)	4.89 (4.27-5.60)	1.82 (0.86-3.88)	3.66 (2.68-5.01)
Personality disorders	6.53 (5.72-7.46)	3.08 (2.63-3.59)	7.71 (3.79-15.70)	1.59 (1.04-2.43)
Previous self-harm events (as cumulative counts)	1.03 (1.02-1.03)	1.04 (1.03-1.06)	NA	NA

Abbreviations: HR, hazard ratio; NA, data not available; NI, variable not included in the final models based on forward selection; Q, quintile; RR, relative rate.

^a^ Akaike information criterion and bayesian information criterion were considered for model selection.

^b^ Both Akaike information criterion and bayesian information criterion suggested the full models to be the best fit for both sexes.

^c^ The model selection in the male and female cohorts both suggested to exclude neighborhood income quintile, and the model selection in females further suggested to exclude rural index; to be consistent across sexes we excluded only neighborhood income quintile in the final models for both sexes.

^d^ Autism diagnosis, as the exposure variable, was always kept in the final models.

### Suicide Death

The suicide death cohort included 334 690 individuals (median age at maximum follow-up, 19 years [IQR, 14-27 years]; median age of first autism diagnosis claim for autistic individuals, 10 years [IQR, 5-16 years]; 17 982 autistic females, 71 928 nonautistic females;48 956 autistic males, 195 824 nonautistic males) followed up for death outcomes from 1993 to 2018 (Table 2). Ascertainment resulted in an older age distribution of the female group than the male group. Over the 25 study years, among autistic people, the cumulative incidence rates of death per 100 000 population were 95 for females and 116 for males for suicide, and 9799 for females and 3413 for males for nonsuicide causes. Among matched nonautistic people, the rates were 46 for females and 83 for males for suicide, and 8114 for females and 2591 for males for nonsuicide causes.

The hazards were higher for autistic females than nonautistic females for suicide death (crude cause-specific HR, 1.98; 95% CI, 1.11-3.56) and nonsuicide death (1.08; 95% CI, 1.03-1.14, as a competing risk), which did not change when adjusted for neighborhood income and rurality (Table 3). Adjusting for each psychiatric diagnosis attenuated most HRs for suicide death to nonsignificant levels. Adjusting for intellectual disabilities increased the HR for suicide death (2.35; 95% CI, 1.28-4.32) but attenuated the HR for nonsuicide death (1.02; 95% CI, 0.96-1.07).

The hazards among autistic males vs nonautistic males were not significantly different for suicide death (crude HR, 1.34; 95% CI, 0.99-1.82), but were significantly higher for nonsuicide death (HR, 1.15; 95% CI, 1.09-1.22) (Table 3). When adjusted for income and rurality, the HR for suicide death became significant (1.36; 95% CI, 1.01-1.85). Adjusting for each psychiatric diagnosis attenuated most HRs for suicide death to nonsignificant levels, even suggesting a reduced risk of suicide death among autistic individuals when adjusted for schizophrenia spectrum disorders (0.69; 95% CI, 0.48-0.99). Adjusting for intellectual disabilities increased the HR for suicide death (1.62; 95% CI, 1.18-2.23) but attenuated that for nonsuicide death (1.00; 95% CI, 0.94-1.06).

The final models (Table 4) showed that, for both females and males, autism per se was not significantly associated with suicide death. Rather, significant correlates among females were intellectual disabilities (associated with reduced risks), mood and anxiety disorders, schizophrenia spectrum disorders, and personality disorders (all associated with increased risks). Significant correlates among males were rurality (rural residence associated with increased risks), intellectual disabilities (associated with reduced risks), and all psychiatric diagnoses (all associated with increased risks). As a substantial proportion (95 085 of 334 690 [28.4%]) of this cohort did not have data on self-harm events, we were unable to examine the association of self-harm event(s) with suicide death in these models.

## Discussion

In this first Canadian population-based study with, to our knowledge, the most recent (diagnosed 1988-2018) and largest cohorts of autistic individuals (self-harm events, 75 926; suicide death, 66 938) among population-based studies to date, we found more self-harm events leading to emergency health care and suicide death among autistic individuals than nonautistic individuals, across males and females. Over 15 years, autistic females showed the highest cumulative self-harm events, followed by autistic males, nonautistic females, and nonautistic males; over 25 years, autistic males had the highest cumulative incidence of suicide death, followed by autistic females, nonautistic males, and nonautistic females. After accounting for neighborhood income and rurality, intellectual disabilities, and psychiatric diagnoses, autism diagnosis was still independently associated with increased self-harm risks, with an 83% increase among females and 47% increase among males. Nevertheless, the increased suicide death risks for both sexes were explained by psychiatric diagnoses, whereas intellectual disabilities attenuated the risks.

Across clinical, community, and registry-based data sets, autistic people had over 3-fold greater odds than nonautistic people of experiencing self-injurious behavior, suicidal ideation, suicide attempt, or suicide death^4^; among autistic people without intellectual disabilities, the meta-analyzed suicidal behavior prevalence was 24.3% (95% CI, 18.9%-29.6%).^8^ For autistic people 25 years of age or younger, pooled prevalence rates were 25.2% (95% CI, 18.2%-33.8%) for suicidal ideation, 8.3% (95% CI, 4.3%-15.6%) for self-harm events or suicide attempts, and 0.2% (95% CI, 0.05%-0.52%) for suicide death.^3^ For self-injurious behavior specifically, the pooled prevalence was 42% (95% CI, 38%-47%) among autistic people^42^ and the cumulative incidence among individuals with developmental disabilities (including autism) was 22% over 1 to 8 years.^5^ Our findings corroborate these previous observations and highlight the importance of evaluating self-harm events and suicide death separately, stratified by sex, and accounting for confounders informing risk mechanisms—particularly psychiatric illnesses—especially because the latest meta-analyses could not summarize their roles owing to the lack of primary data.^4,8^

Our findings of increased self-harm risks among autistic individuals are consistent with population-based findings from Sweden,^15,17^ Denmark,^18^ Finland,^19^ Taiwan,^20^ the UK,^22^ and the US^11,12,13^ that autism diagnosis is independently associated with risk of self-harm or suicide attempts, but the associations of self-harm or suicide attempts with psychiatric diagnoses are substantial and often larger than that of autism. Risks for suicidal behaviors span predisposing factors (eg, genetics, early-life adversity), developmental factors (eg, anxiety, impulsivity, executive dysfunction), and precipitating factors (eg, psychiatric illnesses), as well as social-contextual determinants (eg, social isolation, poor mental health care, access to lethal means).^43^ Some of these factors are frequently experienced by autistic people (eg, self-regulation and executive function difficulties, childhood adversity, inappropriate mental health care, and social disconnection) and are likely associated with the heightened risks of self-harm or suicide attempts.

There are not many population-based studies on suicide death^10,14,15,18,19,21^ and for those assessing the associations with psychiatric diagnoses, our findings corroborate those of studies from Sweden,^15^ Finland,^19^ and Denmark^18^: autism is not independently associated with risk of suicide death after accounting for psychiatric diagnoses, which themselves are substantially associated with increased risks. To our knowledge, our cohort of autistic individuals is the largest to date and likely with the highest statistical power to detect significant associations with autism per se. Psychiatric illnesses, especially substance use disorders, psychotic disorders, and mood disorders, are strong proximal suicide risk factors.^43,44^ Given that psychiatric disorders are more prevalent among autistic people than nonautistic people,^45^ especially in females,^25^ preventing and treating psychiatric illnesses, with sex-specific considerations, are key to offset suicide risks among autistic people.

The presence of intellectual disabilities appears to be associated with increased risk of self-harm but reduced risk of suicide death among autistic people.^14,15,18,19,21^ These seemingly paradoxical findings should be interpreted cautiously considering the multiple pathways of self-harm and suicidal behaviors,^43^ and we caution treating intellectual disabilities as simply a protective factor for suicide death among autistic people. Our findings corroborate those from US-based studies that intellectual disabilities are associated with increased intentional self-harm and suicide attempts, including among autistic people,^11,13^ suggesting that the increased risk is not specific to autistic people without intellectual disabilities. The association of reduced suicide death in the presence of intellectual disabilities might reflect more restrictive access to lethal means and/or receipt of more intense support.

Owing to sex-specific features in suicide-related behaviors^23,24^ and their intersection with autism,^3^ we used sex-stratified analyses rather than arbitrarily controlling for sex. The cumulative incidence of self-harm is highest among autistic females and the cumulative incidence of suicide death is highest among autistic males, comparable with patterns in the general population^23^ and meta-analytic findings among autistic individuals.^42^ The sex-stratified final models show similar patterns of autism-related, sociodemographic, and clinical risks across the self-harm and suicide death outcomes, with descriptively stronger associations with substance use disorders in males, and personality disorders, mood and anxiety disorders in females. This finding implicates certain sex-unique roles of psychiatric diagnoses in self-harm and suicide risks among autistic people.

### Limitations

This study has some limitations. We included all identifiable autistic people in Ontario via an algorithm that is highly specific but moderately sensitive.^28,29^ The autism cohort is hence likely an underestimation (eg, not capturing those diagnosed in private practices or with subtle presentations and yet to be diagnosed) and one biased toward those with more mental health care contacts, partly accounting for the observed increased self-harm and suicide risks. Meanwhile, the misclassification of autistic people in the nonautistic cohort can result in an underestimation of suicide-related outcomes in the autistic cohort. The suicide-related outcomes might be insufficiently sensitive (eg, self-harm not leading to emergency health care was not captured). The rates of intellectual disabilities were lower than those in representative birth cohorts,^46^ implying an underestimation. The sex-stratified design allows for sex-based illustration but prevents direct statistical comparisons between autistic males and females. The total-population design resulted in an older age distribution in the female than the male groups. The administrative data do not reliably identify specific diagnoses that have been shown to be associated with suicide risks, such as other neurodevelopmental diagnoses (eg, attention-deficit/hyperactivity disorder)^11,15,17,18,21^ and subcategories of mood disorders (eg, depressive vs bipolar disorders).^3,6,21^ Finally, the administrative data do not contain other key information about risk and protective mechanisms of suicide behaviors in the general population (eg, race and ethnicity, family history, and social supports),^43,44^ which can be even more critical for autistic people,^3,6^ preventing fine-grained analyses for intersectionality and mechanistic implications.

## Conclusions

In this cohort study, autistic individuals, particularly females, experienced increased risks for self-harm events and suicide death. Across sexes, psychiatric diagnoses significantly accounted for these increased risks, in partially sex-unique ways. Clinical and social support tailored for autistic people to reduce suicide risks should consider multifactorial mechanisms, with a particular focus on the prevention and timely treatment of psychiatric illnesses.

## Notes

Supplement 1. **eTable 1.** ICES Databases Used for This Study **eTable 2.** Diagnostic Codes Used for Ascertaining Intellectual Disabilities and Psychiatric Diagnostic Categories **eAppendix.** Further Explanations to the Cohort Design and Model Assumptions

Supplement 2.
Data Sharing Statement
