# Supplementary material for: Self-Harm Events and Suicide Deaths Among Autistic Individuals in Ontario, Canada
Source: JAMA Netw Open. 2023 Aug 8;6(8):e2327415. doi: 10.1001/jamanetworkopen.2023.27415 (PMC10410481; doi:10.1001/jamanetworkopen.2023.27415)
Supplement: Supplement 1. — eTable 1. ICES Databases Used for This Study eTable 2. Diagnostic Codes Used for Ascertaining Intellectual Disabilities and Psychiatric Diagnostic Categories eAppendix. Further Explanations to the Cohort Design and Model Assumptions [file jamanetwopen-e2327415-s001.pdf]

Supplementary Online Content

Lai MC, Saunders NR, Huang A, et al. Self-harm events and suicide deaths among autistic individuals in Ontario, Canada. *JAMA Netw Open.* 2023;6(8):e2327415. doi:10.1001/jamanetworkopen.2023.27415

eTable 1. ICES Databases Used for This Study
eTable 2. Diagnostic Codes Used for Ascertaining Intellectual Disabilities and Psychiatric Diagnostic Categories
eAppendix. Further Explanations to the Cohort Design and Model Assumptions

This supplementary material has been provided by the authors to give readers additional information about their work.

© 2023 Lai MC et al. *JAMA Network Open.*

eTable 1. ICES Databases Used for This Study

**Database name**	**Description**
Registered Persons Database (RPDB)	The RPDB provides basic demographic information (age, sex, location of residence, date of birth, and date of death for deceased individuals) for those issued an Ontario health insurance number. The RPDB also indicates the time periods for which an individual was eligible to receive publicly funded health insurance benefits and the best-known postal code for each registrant on July 1st of each year. See: https://datadictionary.ices.on.ca/Applications/DataDictionary/Library.aspx?Library=RPDB
Discharge Abstract Database (DAD)	The DAD is compiled by the Canadian Institute for Health Information and contains administrative, clinical (diagnoses and procedures/interventions), demographic, and administrative information for all admissions to acute care hospitals, rehab, chronic, and day surgery institutions in Ontario. At ICES, consecutive DAD records are linked together to form “episodes of care” among the hospitals to which patients have been transferred after their initial admission. See: https://datadictionary.ices.on.ca/Applications/DataDictionary/Library.aspx?Library=DAD
Same Day Surgery Database (SDS)	The SDS is compiled by the Canadian Institute for Health Information and contains administrative, clinical (diagnoses and procedures), demographic, and administrative information for all patient visits made to day surgery institutions in Ontario. The main data elements include patient demographics, clinical data (diagnoses, procedures, physician), administrative data (institution/hospital number etc.), financial data, and service-specific data elements for day surgery and emergency. See: https://datadictionary.ices.on.ca/Applications/DataDictionary/Library.aspx?Library=SDS
National Ambulatory Care Reporting	The NACRS is compiled by the Canadian Institute for Health Information and contains administrative,

© 2023 Lai MC et al. *JAMA Network Open.*

System (NACRS)	clinical (diagnoses and procedures), demographic, and administrative information for all patient visits made to hospital- and community-based ambulatory care centres (emergency departments, day surgery units, hemodialysis units, and cancer care clinics). At ICES, NACRS records are linked with other data sources (DAD, OMHRS) to identify transitions to other care settings, such as inpatient acute care or psychiatric care. See: https://datadictionary.ices.on.ca/Applications/DataDictionary/Library.aspx?Library=NACRS
Ontario Mental Health Reporting System (OMHRS)	The OMHRS is compiled by the Canadian Institute for Health Information and contains administrative, clinical (diagnoses and procedures), demographic, and administrative information for all admissions to adult designated inpatient mental health beds. This includes beds in general hospitals, provincial psychiatric facilities, and specialty psychiatric facilities. Clinical assessment data is ascertained using the Resident Assessment Instrument for Mental Health (RAI-MH), but different amounts of information are collected using this instrument depending on the length of stay in the mental health bed. Multiple assessments may occur during the length of a mental health admission. See: https://datadictionary.ices.on.ca/Applications/DataDictionary/Library.aspx?Library=OMHRS
Ontario Health Insurance Plan Claims Database (OHIP)	The OHIP claims database contains information on inpatient and outpatient services provided to Ontario residents eligible for the province's publicly funded health insurance system by fee-for-service health care practitioners (primarily physicians) and "shadow billings" for those paid through non-fee-for-service payment plans. The main data elements include patient and physician identifiers (encrypted), code for service provided, date of service, associated diagnosis, and fee paid. See: https://datadictionary.ices.on.ca/Applications/DataDictionary/Library.aspx?Library=OHIP

© 2023 Lai MC et al. *JAMA Network Open.*

Ontario Registrar General Vital Statistics—Deaths (ORGD)	The ORGD contains information on all deaths registered in Ontario starting on January 1, 1990. Information on the causes of death (immediate, antecedent, and underlying) recorded on the death certificate are captured. At ICES, a single cause of death variable is derived based on the underlying cause of death if available and, otherwise, the immediate cause of death using the ICD-9 coding system. See: https://datadictionary.ices.on.ca/Applications/DataDictionary/Library.aspx?Library=ORGD

© 2023 Lai MC et al. *JAMA Network Open.*

eTable 2. Diagnostic Codes Used for Ascertaining Intellectual Disabilities and Psychiatric Diagnostic Categories

Clinical category	ICD-9-CM codes (OMHRS DSM-IV) or ICD-10-CM codes (OMHRS DSM-5)	ICD-10-CA codes (DAD/NACRS)	ICD-9 codes (DAD prior to 2002)	Qualifying diagnoses codes (OHIP DXCODE)
**Substance-related and addictive disorders**	AXIS1_DSM4CODE_DISCH1 = 291.x (all 291 codes, excluding 291.82), 292.x (all 292 codes, excluding 292.85), 303.x (all 303 codes), 304.x (all 304 codes), 305.x (all 305 codes).<br>PROVDX_DSM4CODE_ADM1 =4<br>ICD10CMCODE_DISCH1 = F10.x-F19.x, Z72.0.<br>PROVDX_DSM5CODE_ADM1 =16	DX10CODE1 = F55, F10 to F19	'2910' '2911' '2912' '2913' '2914' '2915' '2918' '2919' '2920' '2921' '2922' '2928' '2929' '303' '3030' '3039' '3040' '3041' '3042' '3043' '3044' '3045' '3046' '3047' '3048' '3049' '3050' '3051' '3052' '3053' '3054' '3055' '3056' '3057' '3058' '3059'	291 Alcoholic psychosis, delirium tremens, Korsakov's psychosis, 292 Drug psychosis, 303 Alcoholism, 304 Drug dependence, 305 Tobacco abuse
**Schizophrenia spectrum and psychotic disorders**	AXIS1_DSM4CODE_DISCH1 = 295.x (all 295 codes), 297.x (all 297 codes), 298.x (all 298 codes).<br>PROVDX_DSM4CODE_ADM1 =5<br>ICD10CMCODE_DISCH1 = F20.81, F20.9,	DX10CODE1 = F20 (excluding F20.4), F22, F23, F24, F25, F28, F29, F53.1	'2950' '2951' '2952' '2953' '2954' '2955' '2956' '2957' '2958' '2959' '2970' '2971' '2972' '2973' '2978'	295 Schizophrenia, 297 Other paranoid states, 298 Other psychoses

© 2023 Lai MC et al. *JAMA Network Open.*

	F22, F23, F25, F06.0/1/2, F28, F29. PROVDX_DSM5CODE_ADM1 =2		'2979' '2980' '2981' '2982' '2983' '2984' '2988' '2989'	
**Mood disorders**	AXIS1_DSM4CODE_DISCH1 = 296.x (all 296 codes), 300.4x, 301.13. PROVDX_DSM4CODE_ADM1 =6 ICD10CMCODE_DISCH1 = F06.3, F31, F32, F33, F34. PROVDX_DSM5CODE_ADM1 (provisional) =3, 4	DX10CODE1 = F30, F31, F32, F33, F34, F38, F39, F53.0	'2960' '2961' '2962' '2963' '2964' '2965' '2966' '2967' '2968' '2969' '3004' '3090' '3091' '3110'	296 Manic-depressive psychoses, involutional melancholia, 311 Depressive disorder
**Anxiety disorders**	AXIS1_DSM4CODE_DISCH1 = 300, 300.0x, 300.2x, 300.3x, 308.3x, 309.0x, 309.24, 309.28, 309.3x, 309.4x, 309.8x, 309.9x. PROVDX_DSM4CODE_ADM1 =7, 15 ICD10CMCODE_DISCH1 = F06.4, F40.0x, F40.1x. F40.2x, F41.0x/1x, F41.8x/9x, F93.0, F94.0. PROVDX_DSM5CODE_ADM1 = 5	DX10CODE1 = F40, F41, F42, F43, F48.8, F48.9; F93.1, F93.2	'3000' '3002' '3003' '3083' '3092' '3093' '3094' '3098' '3099'	300 Anxiety neurosis, hysteria, neurasthenia, obsessive-compulsive neurosis, reactive depression
**Personality disorders**	DSM5CODE_DISCH1 = 301, 301.0x, 301.2x, 301.4x, 301.5x, 301.6x, 301.7x, 301.81-3,	DX10CODE1 = F07, F21, F60	'3010' '3012' '3014' '3015' '3016' '3017'	301 Personality disorders

© 2023 Lai MC et al. *JAMA Network Open.*

	301.89, 301.9x 310.1.<br>PROVDX_DSM5CODE_ADM1 = 18<br>ICD10CMCODE_DISCH1 = F06.8, F42.2x, F42.3, F42.4, F42.8, F42.9, F45.2, F63.3.<br>PROVDX_DSM5CODE_ADM1 = 6		'3018' '3019' '3101'	
**Intellectual disabilities**	OMHRS: Q3 = 1 or Q2d (i.e., Axis II) is: F70-F73, F79 or q2d_retired_2016, q2d_retired_2019 start with 317 318 319	ICD9 start with 317, 318, 319; ICD10 start with F70 F71 F72 F73 F79		2 OHIP claim with Dxcode 319

© 2023 Lai MC et al. *JAMA Network Open.*

eAppendix. Further Explanations to the Cohort Design and Model Assumptions

We designed this matched-cohort study to, while ascertaining the entire autistic population in Ontario as much as possible, be able to robustly account for the effects of key demographic factors (hence the matching for age) and use a sex-stratified analysis framework (hence the matching for sex). Although ICES houses data across the entire population of Ontario, the 1:4 autistic:non-autistic age- and sex-matching allows for more robust control of confounding effects than including these demographic factors as covariates during statistical modeling. The choice of the 1:4 matching ratio is based on considering the balance between achieving higher sample sizes to reliably ascertain rare events and computational feasibility (especially for the recurrent event model analyses).

In the first cohort on self-harm event outcomes, we used the Andersen-Gill recurrent event model; this model assumes that, conditional on the covariates, the event and censoring times are independent (i.e., independent censoring assumption). This means that, at a given point in time, individuals who remain under follow-up have the same future risk for the occurrence of the event as those individuals no longer being followed because of censoring. We assumed censoring due to death and due to loss of insurance coverage were at random. In the self-harm event cohort, 93.5% of the individuals survived to the end of follow-up (December 31, 2020), 3.1% were censored due to death, and 3.4% were censored due to loss of insurance coverage. We consider the potential informative censoring introduced by these two types of censoring to be very minor. Compared to the high RRs for self-harm event(s) (9.46, 95% CI 7.83–11.43 for females; 5.38, 95% CI 4.29–6.76 for males), we are confident to assume that these two censorings are non-informative censoring. As a further check, we included suicide death (when data are available, followed up until

© 2023 Lai MC et al. *JAMA Network Open.*

December 31, 2018) as a self-harm event and repeated the crude models, which showed highly comparable RRs (9.42, 95% CI 7.80–11.37 for females; 5.25, 95% CI 4.19–6.58 for males).

In the second cohort on death outcomes, we used cause-specific competing risk models to assess the risk of suicide death, where other causes of death serve as a competing event. Loss of insurance coverage was assumed as non-informative censoring. An individual who dies of other cause is no longer at risk of death due to suicide. This is the reason why we used the competing risk model instead of a conventional Cox model (which treats other causes of death as non-informative censoring events). In biomedical applications, competing risk events may not be independent of the event of interest. For example, individuals with high risk of suicide may also be at an increased risk of other causes of death (e.g., due to shared risk factors or underlying health conditions). In this scenario, a cause-specific competing risk model treats competing events as separate outcomes and focuses on estimating the cause-specific effect for each event separately. The cause-specific hazard of suicide denotes the instantaneous rate of suicide in individuals who have not yet experienced either event (i.e., those who are still alive). The cause-specific competing risk model assumes independent censoring and requires the proportionality assumption, such that the cause-specific hazard ratio is assumed to remain constant over time.

© 2023 Lai MC et al. *JAMA Network Open.*
